# Low-dose eribulin reduces lung metastasis of osteosarcoma *in vitro* and *in vivo*

**DOI:** 10.18632/oncotarget.26536

**Published:** 2019-01-04

**Authors:** Kenta Watanabe, Yoshihiro Yui, Satoru Sasagawa, Kayo Suzuki, Masahiko Kanamori, Taketoshi Yasuda, Tomoatsu Kimura

**Affiliations:** ^1^ Department of Orthopedic Surgery, University of Toyama, Toyama, Japan; ^2^ Research Institute, Nozaki Tokushukai Hospital, Osaka, Japan; ^3^ Department of Human Science, University of Toyama, Toyama, Japan

**Keywords:** eribulin, osteosarcoma, metastasis, LM8, circulating tumor cells

## Abstract

Lung metastasis markedly reduces the prognosis of osteosarcoma. Moreover, there is no effective treatment for lung metastasis, and a new treatment strategy for the treatment of osteosarcoma lung metastasis is required. Therefore, in this study, we investigated the suppressive effect of the microtubule inhibitor eribulin mesylate (eribulin) on lung metastasis of osteosarcoma. At concentrations >proliferation IC_50_, eribulin induced cell cycle arrest and apoptosis in a metastatic osteosarcoma cell line, LM8. However, at concentrations <proliferation IC_50_, (low dose), eribulin changed cell morphology and decreased LM8 migration. Low eribulin concentrations also reduced directionality during migration, peripheral localization of adenomatous polyposis coli protein, and turnover of focal adhesions. In a three-dimensional collagen culture system, low eribulin concentrations inhibited tumor cell proliferation and colony formation. Higher doses of eribulin administered on a standard schedule inhibited lung metastasis and primary tumor growth in a murine osteosarcoma metastasis model. Frequent low-dose eribulin administration (0.3 mg/kg every 4 days × 4) effectively inhibited lung metastasis but had little effect on primary tumor growth. Overall, our results indicate that eribulin could reduce osteosarcoma lung metastasis.

## INTRODUCTION

Osteosarcoma is the most common malignant bone tumor among children and adolescents. The prognosis of osteosarcoma has been poor over the past thirty years. The overall five-year survival rates without and with metastasis at the time of diagnosis are 60–70% and 15–20%, respectively [1, 2] mainly because there are few therapeutic options for lung metastasis of osteosarcoma. The most effective treatment option so far is neoadjuvant chemotherapy developed in the 1980s and consisting of high-dose methotrexate, doxorubicin, and cisplatin (MAP) [3, 4]. Unfortunately, no clinical trials for metastasis inhibition have improved the outcome of patients with osteosarcoma, especially for those who respond poorly to MAP regimens [5, 6]. Therefore, a new treatment strategy for osteosarcoma lung metastasis is required.

Several factors contribute to metastasis from primary tumors to remote organs. Many anti-metastatic agents targeting these processes are under development. Cell migration is a major target for metastasis treatment. It involves tumor cell invasion into surrounding tissues, intravasation into blood vessels, and extravasation into metastatic organs [7]. Metastatic colonization by circulating tumor cells (CTC) has recently emerged as a new target for metastasis treatment. The mechanism of CTC colonization includes the evasion of immune surveillance, adaptation to the metastatic microenvironment, and survival as dormant tumor cells [8]. However, only a few clinical trials on molecular targeted anti-metastasis therapies have demonstrated efficacy. The specific difficulty to find a new treatment strategy for lung metastasis associated with osteosarcoma is that there are few targetable mutations or highly reproducible metastatic murine models available to test anti-metastatic agent efficacy [9].

Microtubule targeting agents (MTAs) are excellent anticancer drugs because they inhibit cell division by suppressing microtubule dynamics [10–12]. Eribulin mesylate (eribulin) is an MTA, which destabilizes microtubules by targeting microtubule plus ends and inhibiting microtubule elongation [13–17]. Like other MTAs, its main anti-tumor actions are the suppression of mitotic spindle organization and the induction of apoptosis. Eribulin also induces mesenchymal-epithelial transition and suppresses cell migration [18–20]. Unlike other anti-microtubule agents that inhibit angiogenesis, eribulin remodels abnormal tumor vasculature. It is used as a second- or third-line treatment for recurrent breast tumor and malignant soft tissue sarcoma. However, the Children's Oncology Group phase II eribulin trial for relapsed osteosarcoma failed, showing no efficacy on tumor shrinkage [21, 22], although eribulin suppressed tumors in osteosarcoma xenografts [23].

There are limited preclinical and clinical data for the suppressive effects of eribulin on metastasis. In preclinical experiments, eribulin only suppressed lung metastasis of a breast cancer cell line in a mouse tail vein injection model [24]. To date, it is unknown how eribulin suppresses metastasis. The results of phase III trials of eribulin for both metastatic breast cancer [25, 26] and advanced soft tissue sarcoma [27] indicated that eribulin prolonged overall survival but seldom extended progression-free survival. These results strongly suggest that eribulin suppresses metastasis progression even when it does not significantly suppress primary tumor growth.

To develop a new treatment option for osteosarcoma lung metastasis, we investigated whether eribulin inhibited lung metastasis of osteosarcoma in a murine metastasis model. Low concentrations of eribulin were anti-metastatic by means other than the induction of cell cycle arrest and apoptosis. Frequent low-dose eribulin administration inhibited lung metastasis of osteosarcoma, indicating the potential of eribulin in the reduction of lung metastasis of osteosarcoma.

## RESULTS

### Inhibition of lung metastasis by eribulin in a murine model

**Figure 1:**
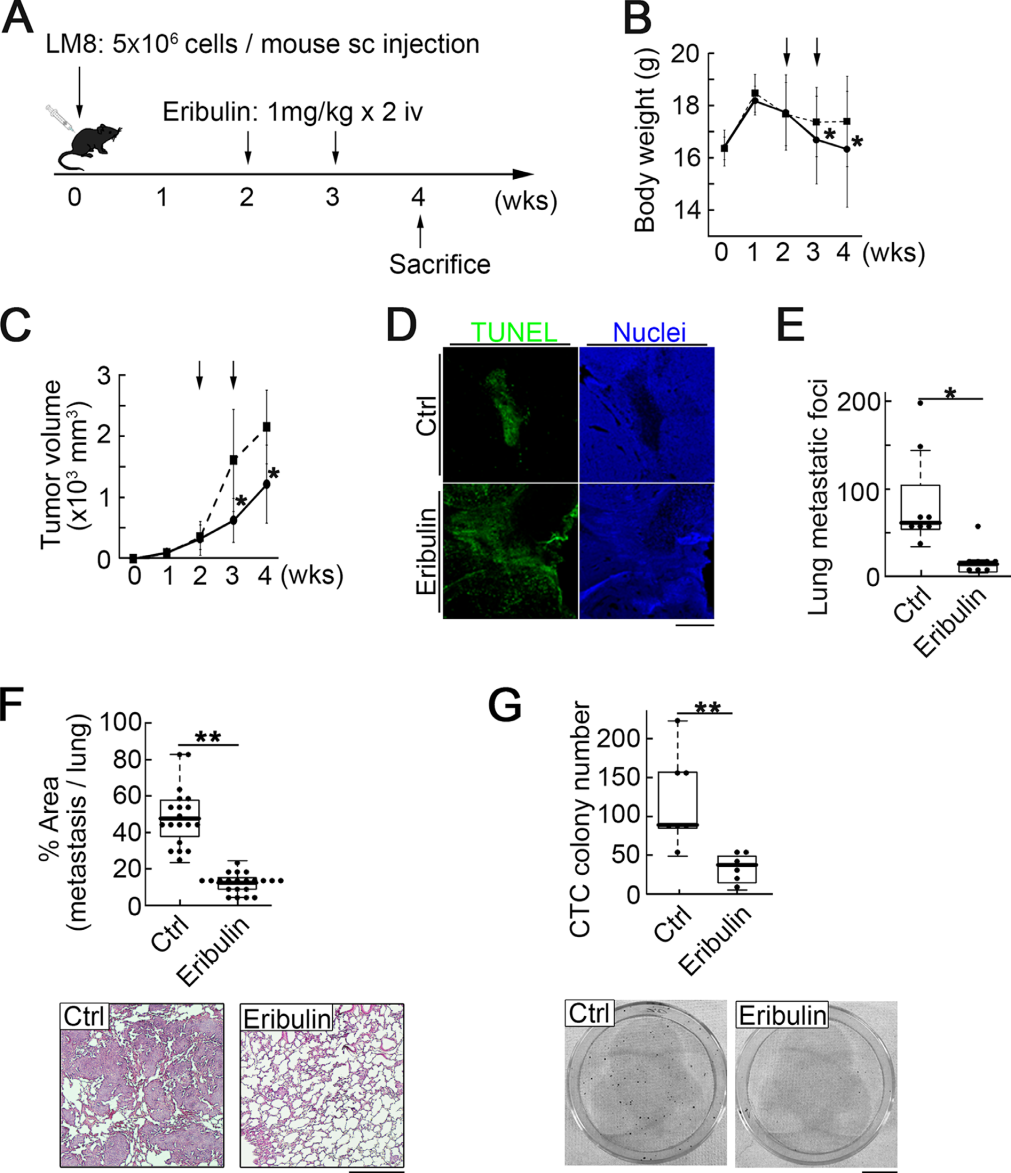
Intravenous eribulin injection in a syngeneic mouse osteosarcoma metastasis model (**A**) Experimental schedule. Sixteen mice were divided into two groups: 1) untreated controls (eight mice); 2) treated with eribulin at 1 mg/kg/week for two weeks (eight mice). (**B**) Body weights of mice in the control (dotted line) and treatment (solid line) groups. Values are mean ± SEM (*n* = 5). ^*^P < 0.05. (**C**) Eribulin inhibited primary tumor growth. Arrows: injection of PBS or eribulin. Values are mean ± SEM (*n* = 5). Solid line: treatment group; dotted line: control group. Tumor volumes were significantly lower in the treatment than in the control group at days 21 and 28. ^*^P < 0.05. (**D**) Representative fluorescence images of primary tumors in the control and treatment groups stained for Tunel (green) and nucleus (blue). Scale bar: 500 *μ*m. (**E**) Number of metastatic foci in the lungs was significantly lower in the treatment than in the control group. Median, quartiles, and highest and lowest values are indicated on the box-and-whisker plots. ^*^P < 0.05. (**F**) Ratio of metastatic area to total lung area was significantly lower in the treatment than in the control group. Median, quartiles, and highest and lowest values are indicated on the box-and-whisker plots (top). Representative H&E-stained sections of pulmonary metastasis in the control (bottom left) and treatment (bottom right) groups are shown. Scale bar: 500 *μ*m. ^**^P < 0.01. (**G**) CTC colony number per 40 *μ*L of peripheral blood. Median, quartiles, and highest and lowest values are indicated on the box-and-whisker plots (top). Images of CTC colonies in the control (bottom left) and treatment (bottom right) groups. Scale bar: 1 cm. ^**^P < 0.01.

We first investigated whether eribulin inhibits osteosarcoma lung metastasis in a mouse model using a clinical administration schedule. According to the package insert, eribulin was clinically administered at 1.4 mg/m^2^ on day 1 and day 8. The pharmacokinetics data for humans and mice revealed that 1 mg/kg eribulin administered to mice had similar pharmacokinetics to 1.4 mg/m^2^ eribulin in humans. Thus, we administered eribulin at 1 mg/kg every 7 days × 2 in the osteosarcoma metastatic model (Figure 1A). The body weights of mice in the treatment group were significantly lower than those in the control group (Figure 1B). Eribulin treatment significantly suppressed primary tumor growth (Figure 1C) and induced apoptosis in tumor cells (Figure 1D). We assessed lung metastasis by counting the metastatic foci (Figure 1E) and measuring their area in tissue sections (Figure 1F top). Eribulin clearly reduced lung metatasis. Histological images showed that in the control group, large metastatic foci infiltrated the lung parenchyma. In contrast, small metastatic foci were solitary within the normal alveolar structure in the treatment group (Figure 1F bottom). To determine whether eribulin reduced CTCs, blood samples were collected and cultured to form colonies. The colony number significantly decreased in the treatment group (Figure 1G) relative to that in the control group. These results indicate that eribulin reduced primary tumor growth and lung metastasis of osteosarcoma.

### Suppression of CTC appearance by the low-concentration phase of eribulin

It has been reported that the pharmacokinetics of eribulin intravenously administered at 1 mg/kg presents as a brief high-concentration phase, which surged to ≥100 nM, followed by a long low-concentration phase, which stabilized at ~10 nM for one week [28, 29]. During the long low-concentration phase, eribulin might be bioavailable enough to inhibit metastasis by suppressing tumor cell survival in the blood. To examine the correlation between CTC appearance and eribulin pharmacokinetics, we conducted a time course analysis of CTC appearance representing the rate of CTC in 40 *μ*L mouse blood (Supplementary Figure 1A). The CTC appearance rate in the treatment group decreased 10 days after eribulin injection but eventually increased to the level observed in the control group (Supplementary Figure 1B). Therefore, the suppression of CTC appearance continued during the long low-concentration phase of eribulin.

### Mitotic arrest and apoptosis by high concentrations of eribulin

**Figure 2:**
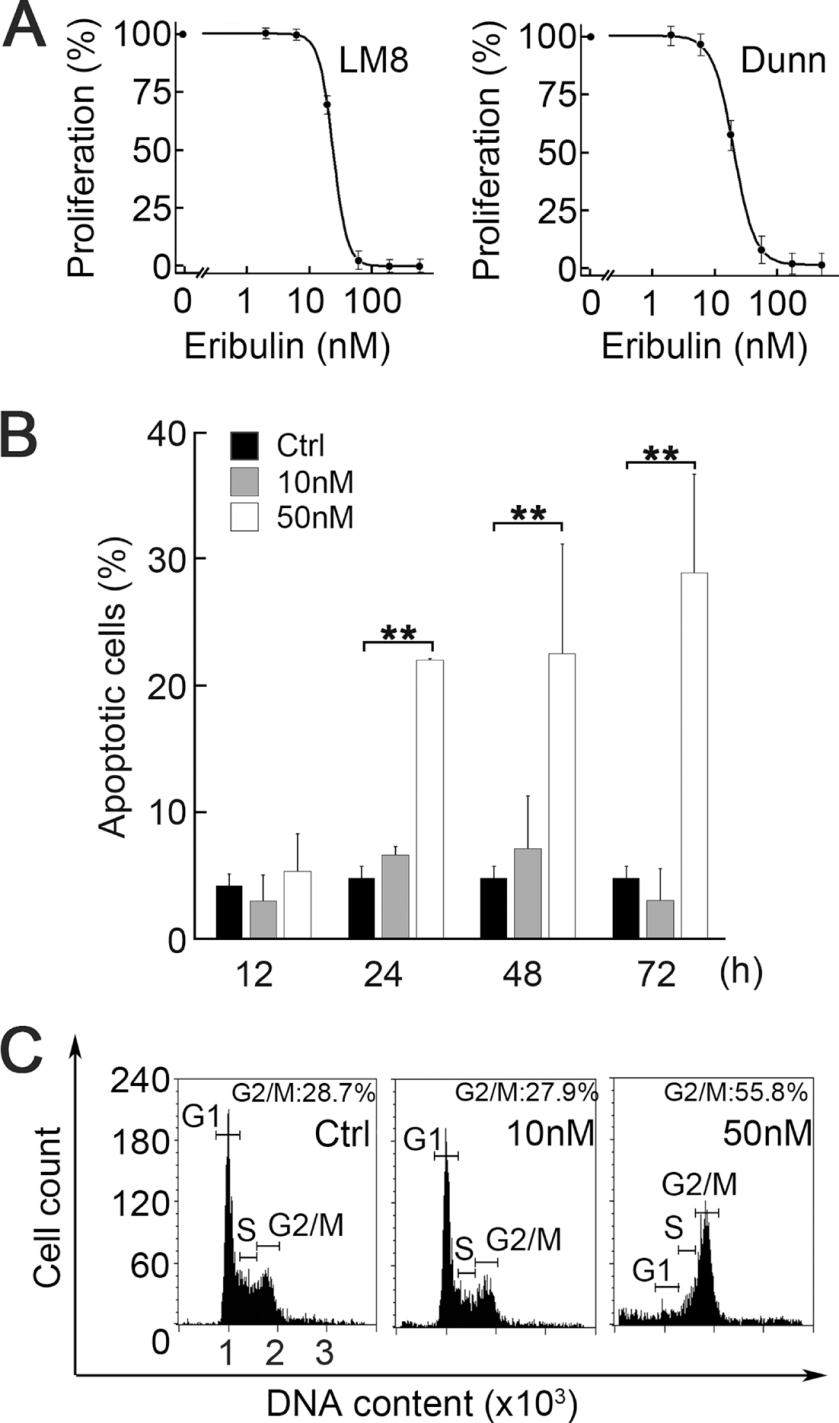
Effect of eribulin on cell proliferation, cell cycle, and apoptosis (**A**) Dose-dependent curves of the proliferation of LM8 cells (left) and Dunn cells (right) exposed to eribulin for 72 h (*n* = 5). IC_50_ of proliferation were 22.8 nM (LM8) and 21.5 nM (Dunn). Values are mean ± SEM (*n* = 3). (**B**) Flow cytometry of apoptosis. LM8 cells were incubated with 0 nM, 10 nM, or 50 nM eribulin for 12 h, 24 h, 48 h, or 72 h. Values are mean ± SEM (*n* = 3). Early apoptosis was induced by 50 nM eribulin. Black: control; gray: 10 nM eribulin; white: 50 nM eribulin. ^**^P < 0.01. (**C**) Representative histograms of flow cytometry for cell cycle distribution. LM8 cells were incubated with 0 nM, 10 nM, or 50 nM eribulin for 12 h. G2/M arrest was induced by 50 nM eribulin.

We speculated that eribulin exerted anti-metastatic action through different mechanisms in the brief high-concentration and long low-concentration phases. We therefore tried to elucidate the contribution of each phase to the reduction of metastasis. We first examined the IC_50_ of proliferation for LM8 and Dunn cells. There was no difference in IC_50_ concentration between the two osteosarcoma cell lines (LM8: 22.8 nM; Dunn: 21.5 nM) (Figure 2A). They were both pharmacokinetically higher than those of the low-concentration phase as well as those previously reported for breast cancer and soft tissue sarcoma [17, 20]. Flow cytometry showed that early apoptosis significantly increased after 24 h treatment with 50 nM eribulin. The slight increase in 7-AAD+/annexin- population with 50 nM eribulin might suggest that eribulin also induced non-apoptotic cell death. On the other hand, apoptosis was not detected with 10 nM eribulin, even after 72 h (Figure 2B; Supplementary Figure 2A). The cell cycle analyses showed that G2/M arrest was induced by 12 h treatment with 50 nM eribulin but not by long-term treatment (72 h) with 10 nM eribulin (Figure 2C; Supplementary Figure 2B). We also examined whether eribulin induced senescence in LM8 cells. No senescence was detected with 12 h treatment with eribulin (Supplementary Figure 3A). Even long-term treatment with eribulin did not induce senescence; LM8 cells only showed senescence in long-term cultures with low serum concentration (Supplementary Figure 3B). We confirmed that eribulin at concentrations >IC_50_ of proliferation induced mitotic arrest and apoptosis in osteosarcoma cell lines. These results were consistent with those previously reported for other cell lines.

### Morphological change and suppression of migration by low concentrations of eribulin

**Figure 3:**
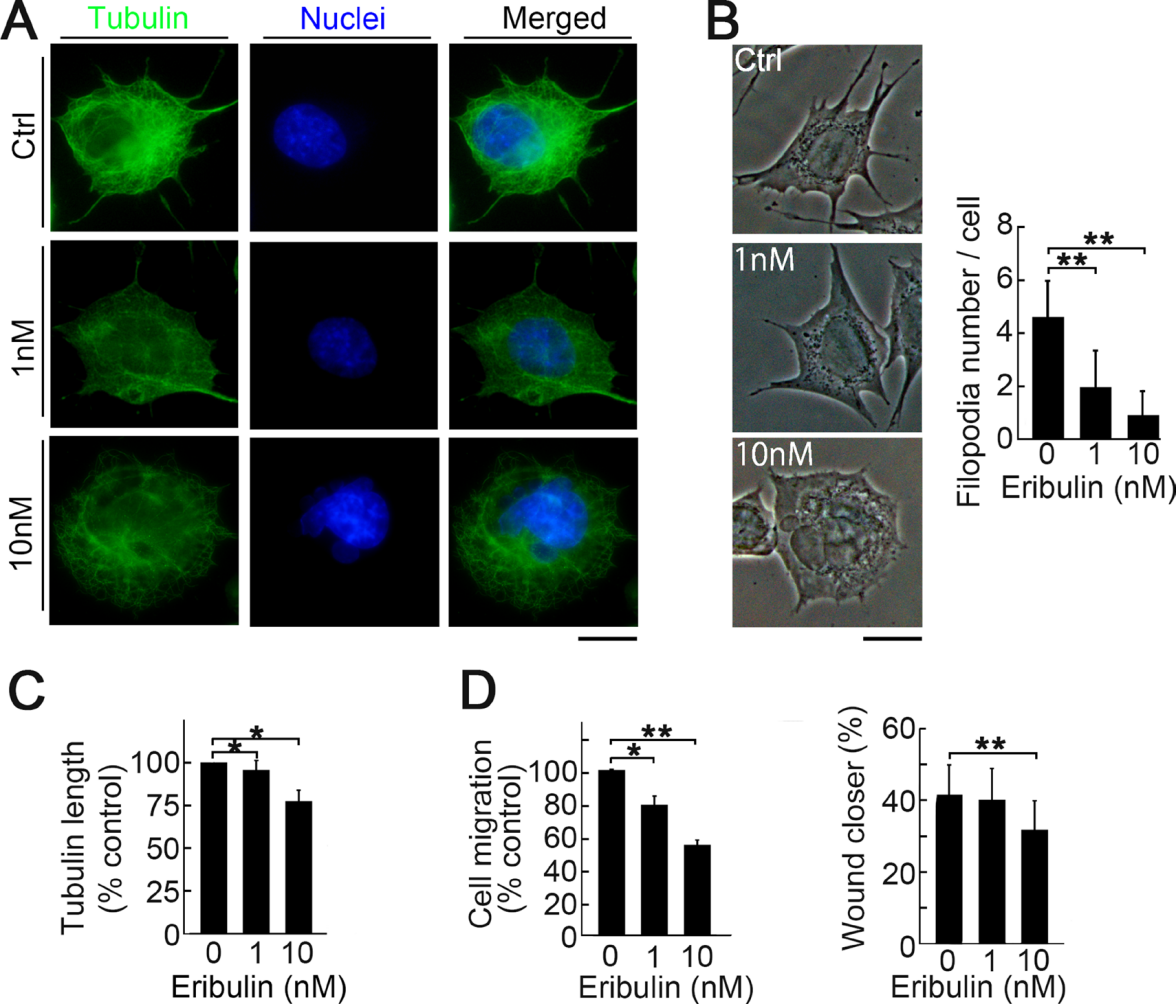
Induction of morphological change and suppression of migration by low eribulin concentrations (**A**) Immunofluorescent images of LM8 cells stained for α-tubulin (green) and nucleus (blue). LM8 cells were treated with eribulin for 16 h. LM8 cells became round and lost their cell protrusions. Scale bar: 10 *μ*m. (**B**) Phase-contrast images showing dose-dependent changes in the morphology of LM8 cells treated with eribulin (left). Number of protrusions on LM8 cells (right). Values are mean ± SEMs (≥30 cells per group). ^**^P < 0.01. (**C**) Length of α-tubulin. Values are mean ± SEM (≥30 cells per group). ^**^P < 0.01; ^*^P < 0.05. (**D**) Percentage of migrated cells for the modified Boyden chamber migration (left) and wound healing (right) assays. Values are mean ± SEM (*n* = 3) ^*^P < 0.05; ^**^P < 0.01. Eribulin suppressed LM8 cell migration in both assays.

We investigated the mechanism by which eribulin at concentrations <IC_50_ of proliferation inhibited metastasis. We focused on cell morphology and motility since important biological signatures of metastatic LM8 cells are their high motility and protrusive morphology [30]. Immunofluorescence imaging showed that low eribulin concentrations cause round cell morphology and reduce protrusions (Figure 3A). Imaging revealed significant decreases in protrusion formation (Figure 3B) and tubulin polymerization (Figure 3C). We also investigated the suppressive effect of eribulin on cell migration using modified Boyden chamber cell migration and wound healing assays. Low eribulin concentrations effectively suppressed cell migration (Figure 3D).

### Reduction of directionality and focal adhesion turnover with low eribulin concentrations

**Figure 4:**
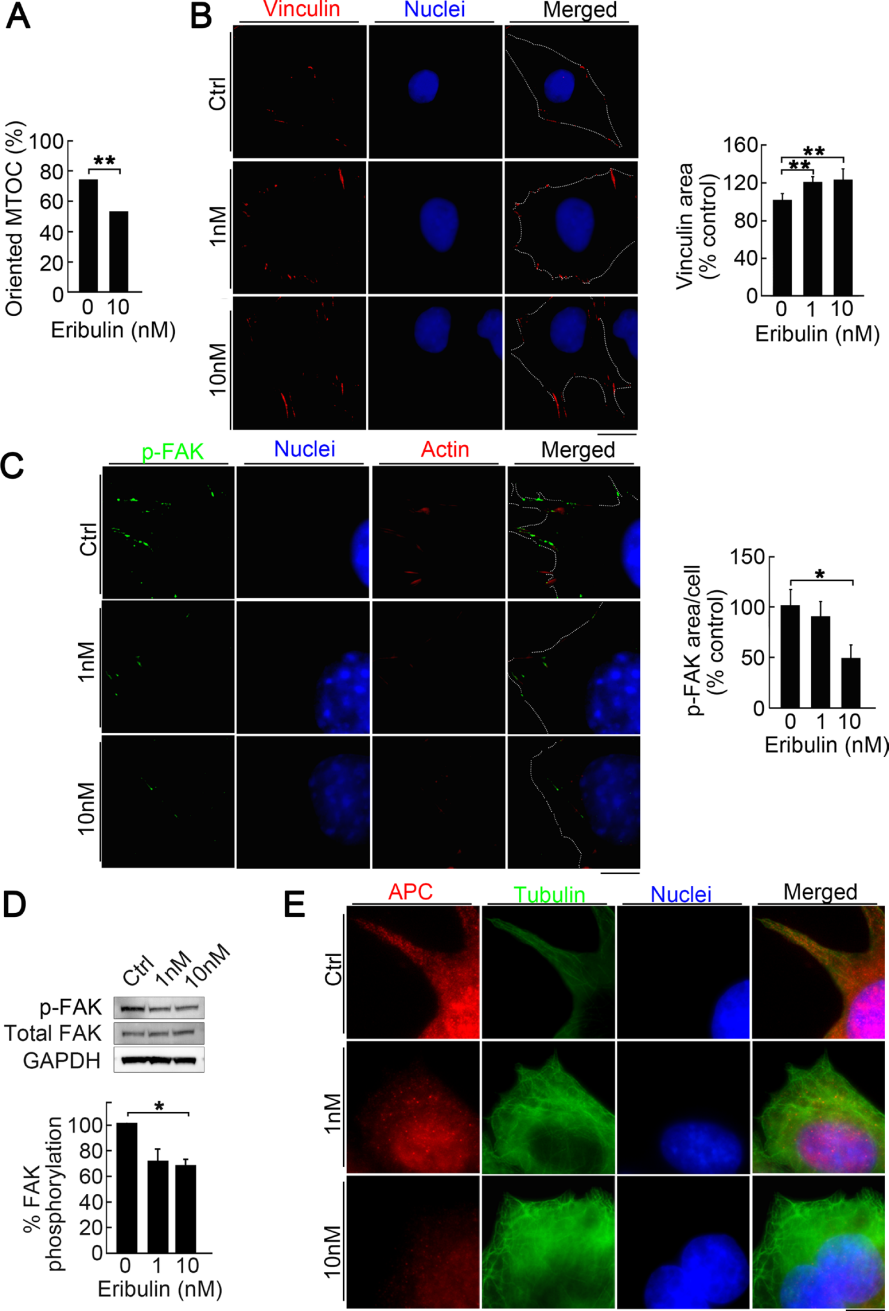
Reduction of directionality and focal adhesion turnover by low eribulin concentrations (**A**) Effect of eribulin on MTOC directionality during wound healing. Percentage of cells with MTOC facing the wound (bottom). Values are mean ± SEM (*n* = 3) ^**^P < 0.01. (**B**) immunofluorescence images of LM8 cells treated with 0 nM, 1 nM, or 10 nM eribulin and stained for vinculin (red) and nucleus (blue) (left). Dotted line shows the cell shape. Scale bar: 10 *μ*m. Quantitative analysis of the area of vinculin staining (right). Values are mean ± SEM (≥30 cells per group). ^**^P < 0.01. (**C**) Immunofluorescence images of LM8 cells treated with 0 nM, 1 nM, or 10 nM eribulin and stained for Tyr397-phosphorylated FAK (green), actin (red), and nucleus (blue) (left). Dotted line shows the cell shape. Scale bar: 10 *μ*m. Quantitative analysis of the area of Tyr397-phosphorylated FAK staining (right). Values are mean ± SEM (≥30 cells per group). ^*^P < 0.05. Eribulin treatment shrank the Tyr397-phosphorylated FAK staining area in a dose-dependent manner. (**D**) Western blot of Tyr397-phosphorylated FAK in LM8 cells treated with eribulin (top). Quantitative densitometric analysis of the ratio of Tyr397-phosphorylated FAK to total FAK (bottom). Values are mean ± SEM (*n* = 3). ^*^P < 0.05. (**E**) Immunofluorescence images of LM8 cells treated with 0 nM or 10 nM eribulin and stained for APC (red), α-tubulin (green), and nucleus (blue). Scale bar: 10 *μ*m.

LM8 cells have higher directionality and activated focal adhesion turnover during migration than Dunn cells [30]. To determine how low eribulin concentrations suppress LM8 migration, we examined cell directionality during wound healing. Cells oriented for migration were defined as those with microtubule-organizing centers (MTOC) localized in the 120° sector facing the wound edge (Supplementary Figure 4). The number of oriented cells was significantly lower in cells for the eribulin treatment than the control (Figure 4A). We also examined the effect of low eribulin concentrations on focal adhesion turnover. Immunofluorescence imaging revealed that eribulin treatment enhanced vinculin staining in a dose-dependent manner (Figure 4B, left). Eribulin significantly increased the area stained by vinculin relative to the control. Therefore, focal adhesions enlarged as focal adhesion turnover decreased (Figure 4B, right). Focal adhesion kinase (FAK) is a key regulator of focal adhesion dynamics and autophosphorylation at the Tyr397 site promotes focal adhesion turnover. LM8 cells in the control group showed strong staining with phosphorylated FAK (pFAK) at the peripheral sites of the cells. Eribulin decreased this staining in a dose-dependent manner (Figure 4C, left). Imaging showed that eribulin significantly reduced pFAK staining at the cell periphery (Figure 4C, right). Reduction of FAK phosphorylation was also confirmed by western blotting (Figure 4D). Recent evidence has shown that three major microtubule-associated proteins—adenomatous polyposis coli (APC), MACF1/ACF7, and cytoplasmic linker-associated proteins (CLASPs)—are involved in microtubule interaction with focal adhesions. Among them, APC is involved in focal adhesion turnover and cell migration [31–35]. Immunofluorescence imaging showed that APC distribution extended to the cell periphery and partially colocalized with focal adhesions in LM8 cells (Figure 4E, top). Eribulin clearly suppressed the peripheral localization of APC (Figure 4E, middle and bottom). The inhibition of APC intracellular transport to the cell periphery by eribulin decreased focal adhesion turnover. At high concentrations, eribulin induced cell cycle arrest and cell death, whereas at low concetrations it reduced cell motility by suppressing focal adhesion turnover and directionality. Both of these contributed to reduced metastasis (Supplementary Figure 5).

### Inhibition of invasion and colony formation in collagen gels by low eribulin concentrations

**Figure 5:**
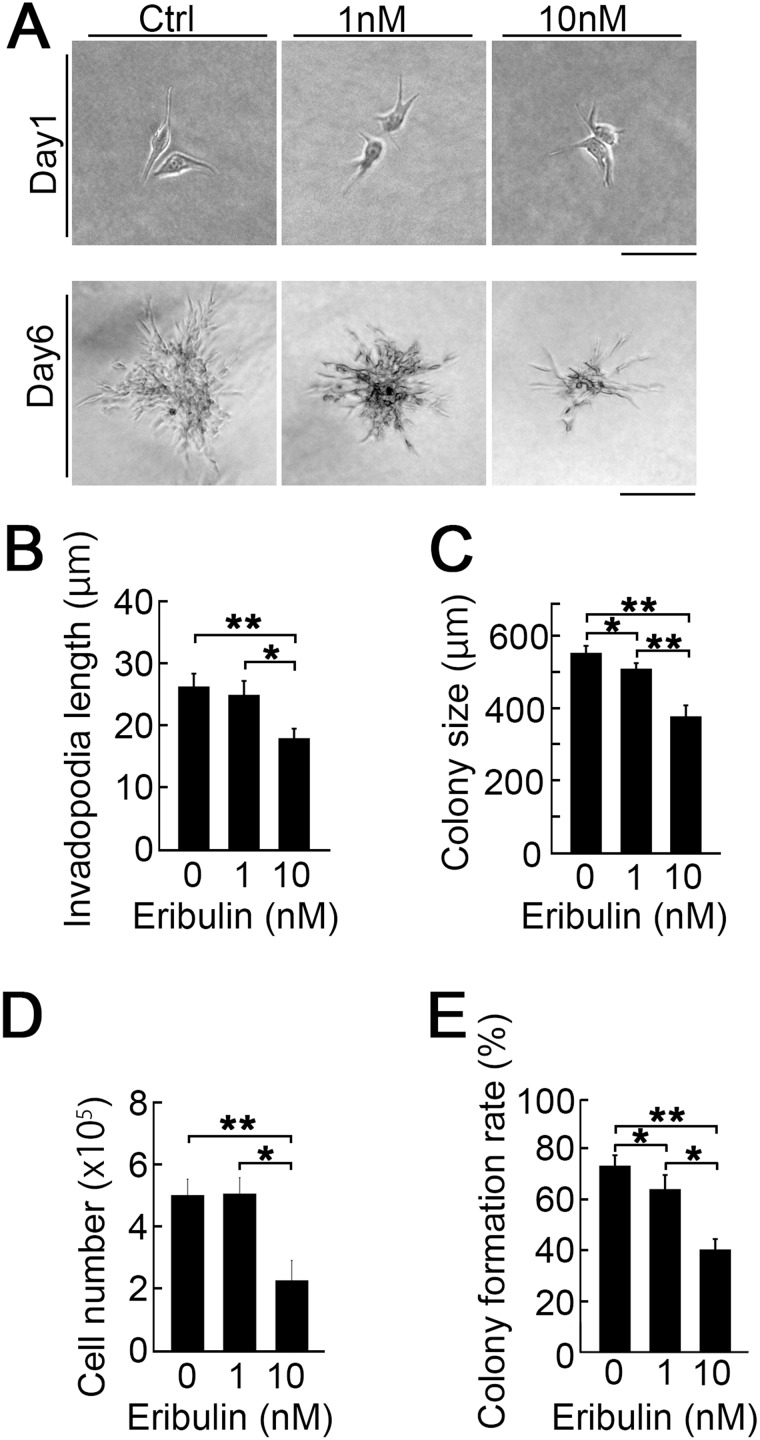
Effect of eribulin on the collagen 3D culture (**A**) Images of LM8 cells in collagen 3D gel (1.5 mg/mL) treated with 0 nM, 1 nM, and 10 nM eribulin on days 1 (top) and 6 (bottom). Scale bars: 40 *μ*m and 100 *μ*m, respectively. (**B**) Quantitative analysis of invadopodia length on day 1. Values are mean ± SEM (≥30 cells per group). Invadopodia shortened in a dose-dependent manner. ^*^P < 0.05; ^**^P < 0.01. (**C**) Quantitative analysis of colony size on day 6. Values are mean ± SEM (≥30 colonies per group). Colony size decreased in a dose-dependent manner. ^*^P < 0.05; ^**^P < 0.01. (**D**) Quantitative analysis of LM8 cell number in collagen 3D gels treated with 0 nM, 1 nM, or 10 nM eribulin. Values are mean ± SEM (≥10 gels per group). ^*^P < 0.05; ^**^P < 0.01. (**E**) Quantitative analysis of colony formation rate (colony number on day 4/applied cell number on day 0). Values are mean ± SEM (≥10 gels per group). ^*^P < 0.05; ^**^P < 0.01.

To mimic the effect of eribulin on metastatic tumor cell engraftment in the soft lung tissue, we used a very soft three-dimensional (3D) collagen culture system. Figure 5A shows invadopodia shrinkage (top) and colony size reduction (bottom) by eribulin. Imaging indicated that eribulin significantly decreased invadopodia length (Figure 5B) and colony size (Figure 5C). We also counted the total cell and colony numbers per gel. At 10 nM, eribulin significantly decreased the total cell number. Therefore, the IC_50_ concentration of eribulin in 3D culture might be lower than that in two-dimensional (2D) culture on a plastic plate (Figure 5D). Low eribulin concentrations also decreased the colony formation rate (colony number on day 4/applied cell number on day 0) (Figure 5E). The decreases in proliferation and colony formation with eribulin in the 3D collagen culture system might reflect reduced metastatic tumor cell engraftment in the lung.

### Frequent low-dose eribulin administration reduced lung metastasis and CTC

**Figure 6:**
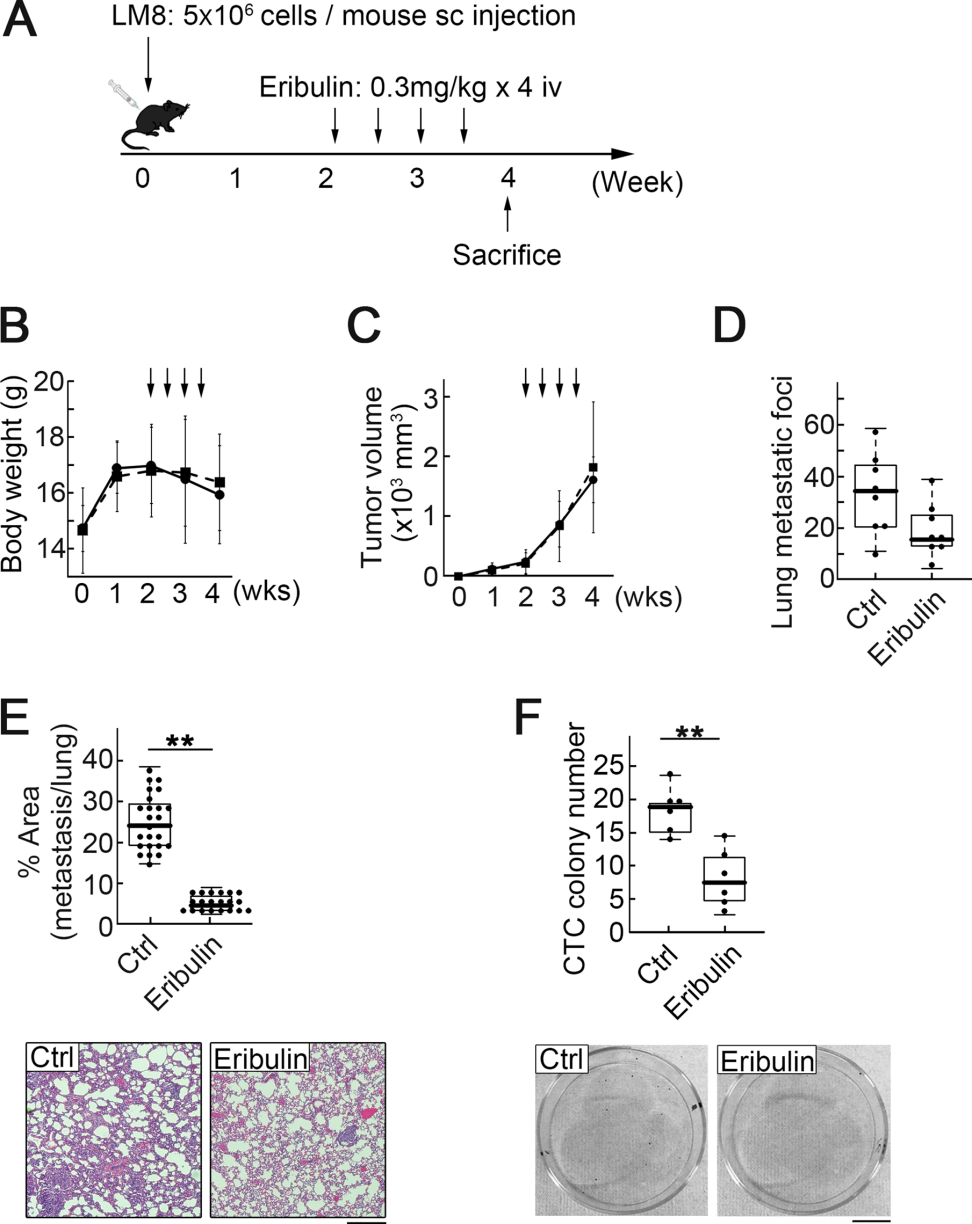
Low-dose eribulin inhibited pulmonary metastasis and CTC colony number (**A**) Experimental schedule. Fifteen mice were divided into two groups: 1) untreated controls (seven mice); 2) models treated with eribulin at 0.3 mg/kg × 2 per week for two weeks (eight mice). (**B**) Body weights of the mice in the control (dotted line) and treatment (solid line) groups. Values are mean ± SEM (*n* = 5). (**C**) Primary tumor growth did not significantly differ between the control and treatment groups. Arrows: injection of PBS or eribulin. Values are mean ± SEM (*n* = 5). Solid line: treatment group; dotted line: control group. (**D**) Number of metastatic foci in lungs was significantly lower in the treatment than in the control group. Median, quartiles, and highest and lowest values are indicated in the box-and-whisker plots. ^*^P < 0.05. (**E**) Ratio of metastasis area to total lung area was significantly lower in the treatment than in the control group. Median, quartiles, and highest and lowest values are indicated in the box-and-whisker plots (top). Representative H&E-stained sections of pulmonary metastasis in the control (bottom left) and treatment (bottom right) groups are shown. Scale bar: 500 *μ*m. ^**^P < 0.01. (**F**) CTC colony number per 40 *μ*L of peripheral blood. Median, quartiles, and highest and lowest values are indicated in the box-and-whisker plots (top). Images of CTC colonies in the control (bottom left) and treatment (bottom right) groups. Scale bar: 1 cm. ^**^P < 0.01.

The anti-metastatic effects of low eribulin concentrations inspired us to explore a new mode of administration with relatively fewer side effects, in which eribulin pharmacokinetics presented a lower peak in the high-concentration phase and a longer duration in the low-concentration phase compared to those observed with the standard treatment schedule. We examined the effect of frequent low-dose eribulin adminstration (0.3 mg/kg every 4 days × 4). The total eribulin dose was reduced to 60% of that of 1 mg/kg every 7 days × 2 (Figure 6A). As expected, frequent low-dose eribulin administration avoided severe body weight loss (Figure 6B). The average size of the primary tumors in the treatment group was similar to that in the control group (Figure 6C). In contrast, frequent low-dose eribulin administration significantly reduced lung metastasis and CTC number to almost the same level as that observed with the standard eribulin treatment schedule (Figure 6D–6F).

## DISCUSSION

In this study, we showed that eribulin was anti-metastatic even at lower concentrations than the IC_50_ of proliferation. Low eribulin concentrations suppressed cell migration, decreased focal adhesion turnover, and reduced colony formation in 3D collagen gels. It is widely accepted that increased tumor cell migration followed by increased CTCs in blood accelerate metastasis progression [7]. We have also reported that increased cell migration and CTCs correlate with osteosarcoma lung metastasis [30, 36]. The suppressive effects of low eribulin concentrations on these mechanisms may have led to metastasis reduction. We confirmed that frequent low-dose eribulin administration reduced lung metastasis but did not inhibit primary tumor growth.

Studies of the anticancer action of microtubule inhibitors have focused mainly on mitotic arrest and cell-killing effects [10–12]. The reduction of lung metastasis by frequent low-dose eribulin administration suggests that the development of microtubule targeting agents as metastatic inhibitors should focus on cell migration, directionality, and tumor vasculature modulation rather than mitosis. Eribulin has a novel anti-migratory mechanism through APC retention in the cell center. Anti-microtubule agents are also anti-angiogenic but their mode of action in this capacity has not yet been elucidated. Nonetheless, eribulin may remodel tumor vasculature and reduce hypoxia in the tumor microenvironment [18]. In the present study, however, we could not determine the effects of eribulin on tumor vasculature because our murine osteosarcoma model seldom showed any vasculature in the primary tumors.

Recent evidence has shown that alternative treatment strategies that administer minimum doses of drugs to maintain tumor stability (metronomic therapy or adaptive therapy) might be more effective for patient survival with metastatic disease rather than the traditional treatment targeting maximal cell death [37–39]. The clinical use of eribulin is limited to single agent administration for a short period due to the occurrence of side effects. Our results showed that frequent low doses of eribulin inhibited metastasis without inducing any severe side effects. Therefore, eribulin has cytostatic action and future metronomic chemotherapies could include it. Combined with standard adjuvant chemotherapies, low doses of eribulin may have a long-term effect of reducing lung metastasis of osteosarcoma and may prolong the survival of osteosarcoma patients who do not have lung metastasis at first diagnosis but later develop lung metastasis. Moreover, frequent low-dose eribulin administration reduced lung metastasis but did not shrink primary tumors. Our results suggest that eribulin could be used in anti-metastatic treatments for other tumors with high IC_50_ concentrations against eribulin but not heretofore indicated for eribulin treatment.

Few new agents evaluated in phase II studies have shown any efficacy in patients with refractory osteosarcoma. One possible explanation for these disappointing outcomes is that most phase II studies evaluate drug efficacy by primary tumor radiography, and calcification masks primary tumor shrinkage [5]. Our results show that the tumor cells at the metastatic site responded differently to anticancer agents than those in primary tumors because of the heterogeneity of the tumor cells, microenvironments, and drug delivery systems between primary- and metastatic sites. New preclinical and phase II evaluations focusing on metastasis reduction are needed to advance progress in osteosarcoma treatment. The anti-metastatic effects of other microtubule-targeting agents, such as taxanes, paclitaxel, and docetaxel, on osteosarcoma lung metastasis should be assessed despite the fact that they have proven ineffective in osteosarcoma phase II studies [40, 41].

Our study has several limitations. We used C3H mice for our syngeneic metastasis model, however, the pharmacokinetics data we referred to in this study were measured using different strains (BALB/c and CF-1). We assume that the pharmacokinetics of eribulin in C3H mice is similar to that in other strains as most eribulin is excreted into the bile and urine without being metabolized after intravenous injection. Sampson et al. reported that some osteosarcoma cell lines regrew in the presence of eribulin, but remained sensitive to eribulin [42]. We did not examine the delayed growth of LM8 cells at metastatic sites. It is possible that lung metastasis of LM8 proliferate later during long-term administration of eribulin, therefore, we need to examine a combination strategy with other chemotherapeutic agents.

Collectively, eribulin is a potential therapeutic option for lung metastasis of osteosarcoma. Its anti-metastatic effects at concentrations lower than IC_50_ suggest that it has a wide application range. Frequent low-dose eribulin administration can reduce lung metastasis for a long time with relatively few side effects.

## MATERIALS AND METHODS

### Ethics statement

This study was conducted in accordance with the ethical standards of the Declaration of Helsinki and according to national and international guidelines and has been approved by the institutional animal experiments review committee (protocol number 29-3-#2).

### Cell culture

Mouse osteosarcoma cell lines LM8 and Dunn were kindly provided by T. Ueda (Osaka National Hospital, Osaka, Japan). The highly metastatic osteosarcoma cell line LM8 was derived from the non-metastatic murine osteosarcoma cell line Dunn osteosarcoma by repeating eight cycles of the procedure described by Poste and Fidler [43]. The cells were maintained in Dulbecco's Modified Eagle's Medium (DMEM) supplemented with 10% fetal bovine serum (FBS) and cultured at 37°C in a fully humidified incubator under 5% CO_2_. All cells were passaged for one month (<15 passages) for the *in vitro* and *in vivo* experiments. All cells were routinely tested for *Mycoplasma* contamination with the CycleavePCR Mycoplasma Detection Kit (TaKaRa Bio Inc., Kusatsu, Shiga, Japan). None of the cell lines used in this article are listed in the International Cell Line Authentication Committee or the NCBI biosample database of misidentified cell lines.

### Antibodies and reagents

Eribulin was obtained from Eisai Co. Ltd. (Tokyo, Japan) through a Material Transfer Agreement. Anti-α-tubulin (DM1A, ab7291), anti-pericentrin (ab4448), and anti-Tyr397-phosphorylated FAK (p-FAK, EP2160Y, ab81298), Goat Anti-Mouse IgG (Alexa Fluor**^®^** 488, ab150113), Donkey Anti-Rabbit IgG (Alexa Fluor**^®^** 555, ab150062), and Donkey Anti-Mouse IgG (Alexa Fluor**^®^** 555, ab150110) were purchased from Abcam (Cambridge, UK). Anti-Vinculin (hVIN-1, NB600-1293) was purchased from Novus Biologicals (Littleton, CO, USA). Anti-APC (F-3, sc-9998) and anti-GAPDH (sc-32233) were purchased from Santa Cruz Biotechnology, Inc. (Dallas, TX, USA). Anti-FAK (#3285) was purchased from Cell Signaling Technology, Inc. (Danvers, MA, USA). Hoechst33342 was purchased from Invitrogen (Carlsbad, CA, USA). Rhodamine-Phalloidin (PHDR1) was purchased from Cytoskeleton, Inc. (Denver, CO, USA). Fibronectin solution (C-43050) was purchased from PromoCell GmbH (Heidelberg, Germany). Horseradish peroxidase (HRP)-anti-mouse IgG and HRP-anti-rabbit IgG were purchased from Jackson ImmunoResearch (West Grove, PA, USA).

### Animal experiments

Four-week-old C3H/HeN mice were purchased from CLEA Japan, Inc. (Tokyo, Japan). LM8 cells metastasize to the lungs by both subcutaneous and intraosseous transplantation. We chose subcutaneous transplantation because of its high reproductivity of the metastatic process, such as CTC appearance and lung metastasis formation, compared to intraosseous transplantation. For the eribulin metastasis reduction experiments, LM8 cells (5 × 10^6^ per mouse) were injected into the subcutaneous tissue of the backs of syngeneic mice. The animals were randomized into control and treatment groups. Eribulin was injected into the tail veins at 1 mg/kg every 7 days × 2 or 0.3 mg/kg every 4 days × 4. Histological evaluations and pulmonary metastasis foci counts were performed four weeks after tumor cell injection because tumor-bearing mice die from lung metastasis around 5 weeks after subcutaneous transplantation. Lungs were fixed with 10% formalin, embedded in paraffin, cut into 8-*μ*m sections, and stained with hematoxylin and eosin (H&E). Apoptosis in the primary tumors was assessed by a TUNEL assay of the paraffin sections using an *In Situ* Cell Death Detection Kit (No. 11684795910; Roche Diagnostics, Risch-Rotkreuz, Switzerland) according to the manufacturer's instructions.

For CTC quantification, eribulin was injected into the tail veins at a rate of 1 mg/kg two weeks after LM8 transplantation as mentioned above. Peripheral blood samples (40 *μ*L) were collected from the tail veins and maintained in DMEM supplemented with 10% FBS and penicillin (100 U/mL)-streptomycin (100 *μ*g/mL) to form colonies as previously described [36]. Colonies were fixed with 10% formalin, stained with crystal violet, and counted.

### Cell proliferation assay

Cell viability was assessed with a CCK-8 WST assay kit (Dojindo Molecular Technologies, Inc., Kumamoto, Japan) according to the manufacturer's instructions. In brief, LM8 cells were plated in 96-well plates at 1 × 10^4^ cells per well and incubated in DMEM with 10% FBS for 24 h. Then, the cells were treated with various eribulin concentrations for 72 h. CCK-8 reagent was then added to the medium, and the cells were further incubated for 2 h at 37°C. Absorbance was measured at 450 nm on a plate reader (Sunrise Rainbow; Tecan Japan Co. Ltd., Kawasaki, Japan).

### Flow cytometry analysis

The cells were plated in 24-well plates at a concentration of 3 × 10^5^ cells per well and were grown for 24 h. The cells were then treated with the indicated eribulin concentrations for the specified time periods. They were stained with Annexin-V and 7-aminoactinomycin D (7-AAD) to identify apoptosis and stained with propidium iodide for cell cycle analysis. The samples were analyzed in a Guava EasyCyte Plus Flow Cytometry System (Merck Millipore, Billerica, MA, USA).

### Immunofluorescence

LM8 cells (1 × 10^3^) were grown on eight-well culture slides (TOHO Corp, Tokyo, Japan) coated with fibronectin (10 *μ*g/mL) and incubated for 24 h. The cells were then treated with eribulin for 16 h. They were fixed with 4% paraformaldehyde for 20 min and permeabilized with either 0.1% Triton X-100 (APC and p-FAK staining) or 0.1% Tween 20 (α-tubulin staining) for 10 min followed by blocking with 0.1% bovine serum albumin (BSA) and 0.1% Tween 20 for 60 min at room temperature. For vinculin staining, the cells were permeabilized at 4°C for 1 min with ice-cold permeabilization buffer (pH 6.9; 10 mM HEPES, 50 mM NaCl, 3 mM MgCl_2_, 0.5% Triton X-100, 300 mM sucrose, and 1 mM EGTA) before fixation. The cells were stained with primary antibodies (α-tubulin 1:500; p-FAK 1:250; APC 1:250; vinculin 1:50) overnight at 4°C followed by secondary antibody staining and counterstaining with Hoechst 33342 for 30 min at room temperature. For F-actin staining, the cells were incubated with rhodamine-phalloidin according to the manufacturer's instructions.

### Microscopy

Cells and tissues were viewed under a phase contrast microscope (Eclipse Ti; Nikon Instruments, Melville, NY, USA) fitted with a Plan Fluor ×40 objective lens (NA 0.6; Nikon Corp., Tokyo, Japan) for tissue sections and a Plan Fluor ×100 objective lens (NA 1.30, oil, Nikon Corp.) for immunofluorescence. Images were processed and analyzed in Fiji/ImageJ v. 2.0.0-rc-67/1.52c (NIH, Bethesda, MD, USA). To quantify microtubule polymerization, we assumed that the length of fiber stained with tubulin represented the length of microtubules. Images stained with anti-α-tubulin antibody were filtered with Bandpass Filter (ImageJ/Fiji plugin) and binarized with Skeletonize (ImageJ/Fiji plugin). The lengths of the skeletonized microtubule images were measured with the AnalyzeSkeleton (2D/3D) (ImageJ/Fiji plugin). Tubulin was considered to be polymerized when the average branch length was >1.6 *μ*m. Areas stained with viculin or phosphorylated FAK were measured by subtracting the immunofluorescent background with a rolling ball and binarizing the images. Regions > 0.03 *μ*m^2^ were measured.

### Protrusion measurement

LM8 cells were plated in eight-well culture slides at a rate of 1 × 10^3^ cells per well and grown for 24 h. The cells were treated with eribulin and incubated for 16 h. Microspikes >20 *μ*m long were defined as protrusions. More than 150 cells from three biological replicates were analyzed.

### Migration assay

The modified Boyden chamber migration and wound healing assays were performed as described previously [44, 45]. In brief, the modified Boyden chamber migration assay was performed for 12 h in 24-well Bio-Coat cell migration chambers (BD Biosciences, Franklin Lakes, NJ, USA). The lower surface of the membrane was coated with 30 *μ*g/mL fibronectin for haptotactic migration. LM8 cells were applied to the upper chamber at a rate of 1 × 10^4^ cells with eribulin treatment for 12 h. Non-migratory cells were removed from the upper surface with cotton swabs. Migrated cells were fixed in 70% v/v methanol, stained with crystal violet, and counted. For the wound healing assay, confluent LM8 cells were scratched and throughly washed with phosphate buffered saline (PBS) to remove detached cells and debris. They were then treated with eribulin for 12 h. Images of wounds were measured with Fiji/ImageJ. Cell directionality was evaluated by fixing the cells during wound healing with 100% ice-cold methanol for 10 min at room temperature then blocking them for 30 min with PBS containing 1% BSA and 0.1% Tween 20. MTOCs were stained with anti-pericentrin antibody (1:1,000) at 4°C overnight. The cells were then incubated with anti-Rabbit IgG (Alexa Fluor**^®^** 555) and Hoechst for 30 min at room temperature. More than 100 cells from three separate experiments were analyzed.

### 3D collagen culture

Collagen gels were fabricated by diluting 3.8 mg/mL acid-solubilized rat-tail collagen (Corning Inc., Corning, NY, USA) in DMEM to 1.5 mg/mL and neutralizing to pH 7.4 with 1 mol/L NaOH. Cell suspensions were added to the wells at a density of 200 cells/well then immediately transferred to a 37°C incubator for 30 min to initiate polymerization. The collagen gels were then covered with culture media containing eribulin. Cells or colonies were observed under a microscope on days 1, 4, and 6. Invadopodia lengths and colony sizes were analyzed using the Fiji/ImageJ software. Colony formation rates were calculated as the colony numbers on day 4 per applied cell number.

### Statistical analysis

Pairs of groups were compared using a two-tailed unpaired Student's *t*-test. Three groups were compared by one-way ANOVA. Comparisons between groups were made with a Tukey-Kramer multiple comparisons test. All statistical analyses were performed with the Statcel v. 3 (OMS Publishing Inc., Tokyo, Japan) add-in for Microsoft Excel (Microsoft Corp., Redmond, WA, USA). Values <0.05 were considered statistically significant. Error bars for all figures represent the standard error of the mean (SEM), unless otherwise stated. Data for *in vitro* experiments represent ≥3 independent biological experiments.

## SUPPLEMENTARY MATERIALS FIGURES AND TABLES



## Abbreviations

CTC: circulating tumor cell
MTA: microtubule targeting agent
MTOC: microtubule-organizing center
FAK: focal adhesion kinase
APC: adenomatous polyposis coli
3D: three-dimensional
2D: two-dimensional
DMEM: Dulbecco's Modified Eagle's Medium
FBS: fetal bovine serum
BSA: bovine serum albumin
PBS: phosphate buffered saline.
