# Supplementary material for: Low-dose eribulin reduces lung metastasis of osteosarcoma in vitro and in vivo
Source: Oncotarget. 2019 Jan 4;10(2):161–74. doi: 10.18632/oncotarget.26536 (PMC6349434; doi:10.18632/oncotarget.26536)
Supplement: Supplementary file 1 [file oncotarget-10-161-s001.pdf]

Low-dose eribulin reduces lung metastasis of osteosarcoma *in vitro* and *in vivo*

SUPPLEMENTARY MATERIALS

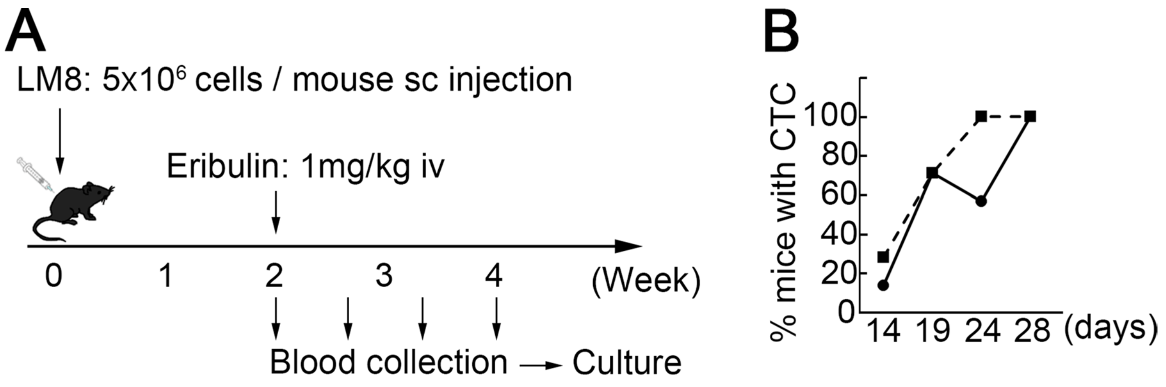

Supplementary Figure 1: (**A**) Schedule of time course analysis of CTC. (**B**) Percentage of mice presenting with CTC in 40 μL of blood. Solid line: treatment group; dotted line: control group.

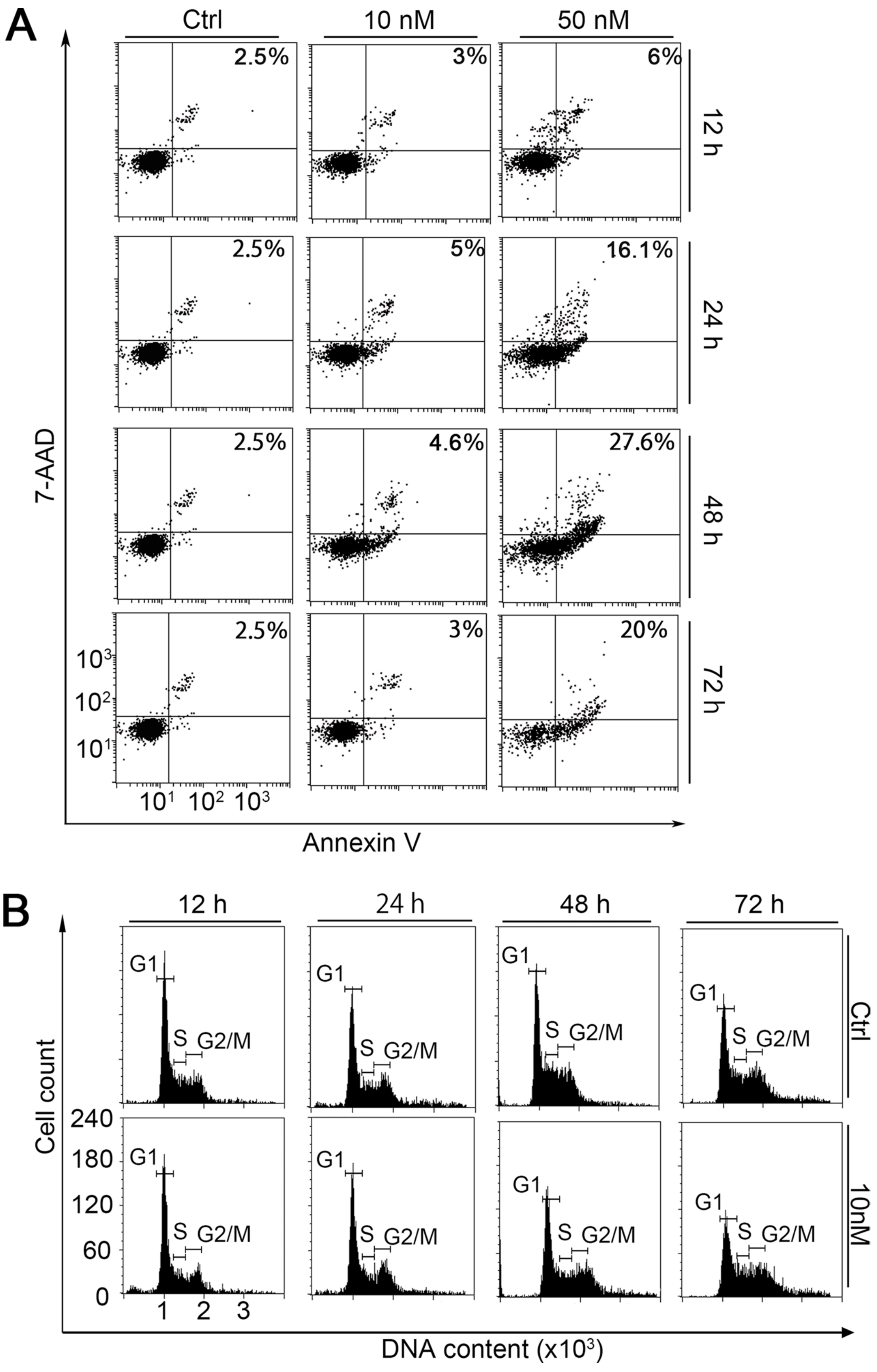

Supplementary Figure 2: (**A**) Representative histograms of flow cytometry for apoptosis. LM8 cells were incubated with 0 nM, 10 nM, 50 nM, or 100 nM eribulin for 12 h, 24 h, 48 h, or 72 h. Early apoptosis increased in LM8 cells after 12 h treatment with 50 nM eribulin relative to the control. (**B**) Histograms of flow cytometry for cell cycle distribution. LM8 cells were incubated with 0 nM or 10 nM eribulin for 12 h, 24 h, 48 h, or 72 h. No G2/M arrest was induced by the 10 nM eribulin treatment.

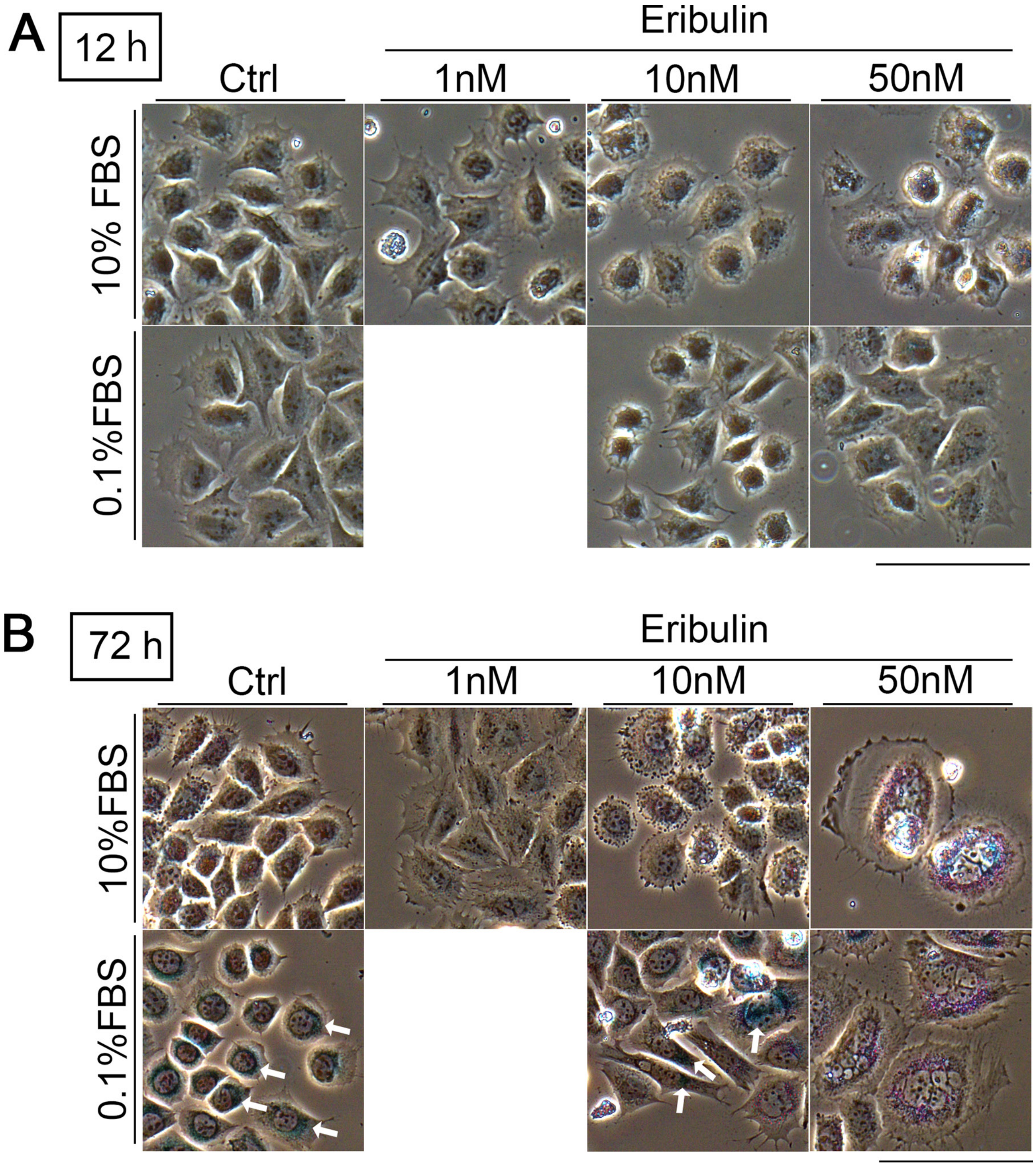

Supplementary Figure 3: (**A**) SA β-Gal staining images. LM8 cells were incubated with 0 nM, 1 nM, 10 nM, or 50 nM eribulin for 12 h in DMEM with 10% FBS (top) and 0.1% FBS (bottom). No senescence was induced for 12 h treatment. Scale bar: 100 μm. (**B**) SA β-Gal staining images. LM8 cells were incubated with 0 nM, 1 nM, 10 nM, or 50 nM eribulin for 72 h in DMEM with 10% FBS (top) and 0.1% FBS (bottom). LM8 cells treated in DMEM with 0.1% FBS rarely showed senescence. Scale bar: 100 μm.

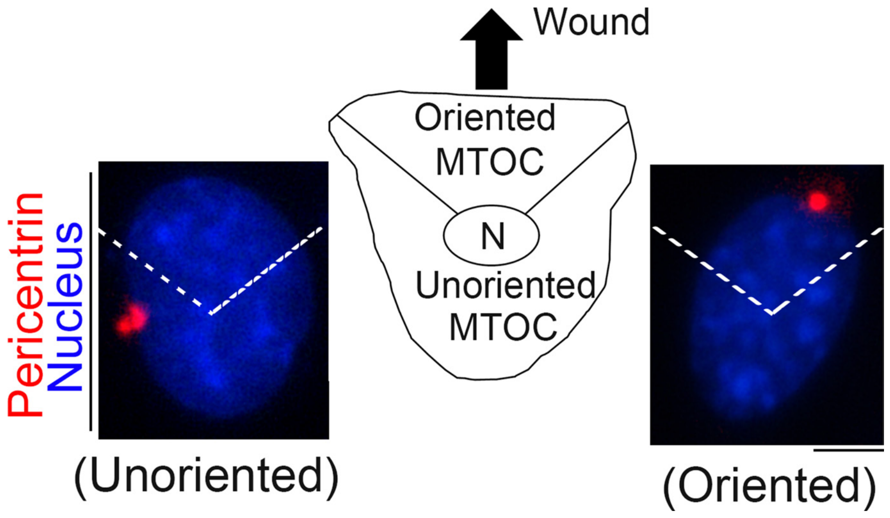

Supplementary Figure 4: Immunofluorescence images of LM8 cells in the wound healing assay and stained for pericentrin (red) and nucleus (blue). Scale bar: 10 μm. Cells in which the MTOC was within 120º facing the wound were scored as positive.

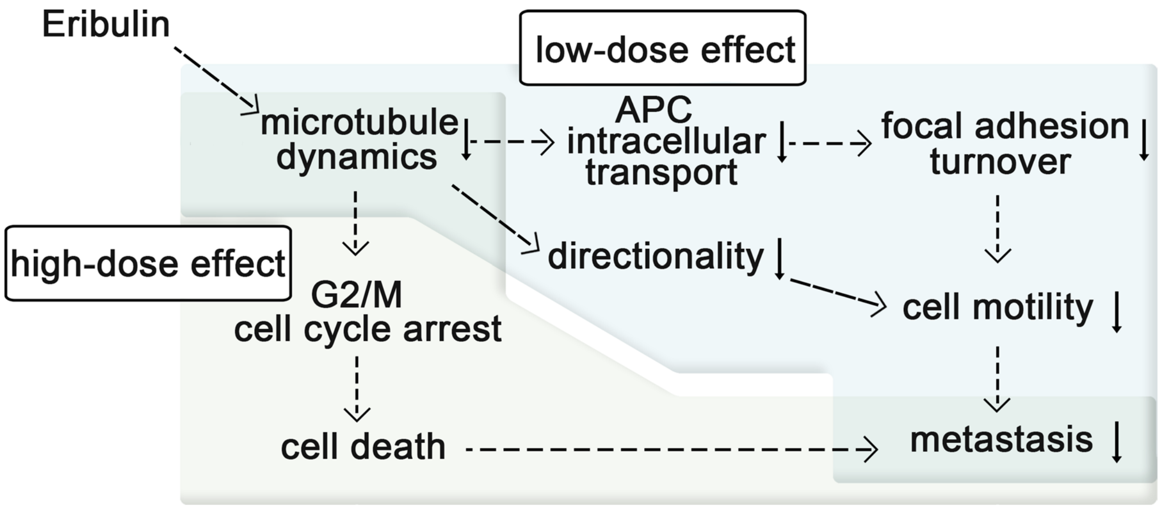

Supplementary Figure 5: Model of the mechanism of eribulin metastasis suppression. Inhibition of microtubule dynamics by high concentrations of eribulin was followed by cell cycle arrest and apoptosis induction. Low concentrations of eribulin suppressed cell migration by reducing focal adhesion turnover and cell polarity. Eribulin retained APC at the cell center which, in turn, might reduce focal adhesion turnover.
